# Knowledge, attitude, motivation and planning of breastfeeding: a cross-sectional study among Jordanian women

**DOI:** 10.1186/s13006-020-00303-x

**Published:** 2020-07-01

**Authors:** Wasim Khasawneh, Khalid Kheirallah, Mai Mazin, Sanaa Abdulnabi

**Affiliations:** 1 Department of Pediatrics and Neonatology, Jordan University of Science and Technology, Irbid, Jordan; 2 Department of Public Health, Jordan University of Science and Technology, Irbid, Jordan

**Keywords:** Breastfeeding, Breastfeeding knowledge, Breastfeeding attitude, Breastfeeding planning

## Abstract

**Background:**

In Jordan, the rate of exclusive breastfeeding is declining. The trend variation in breastfeeding practice is determined by different factors including antenatal women’s attitude and planning which are affected by their awareness and the support they receive. This study aims to assess knowledge, attitude, support, and planning of breastfeeding among Jordanian women.

**Methods:**

A face-to-face cross-sectional semi-structured questionnaire survey was conducted among healthy women in the antenatal clinic and postpartum ward at three hospitals in Northern Jordan during the period August 2019 to December 2019. Data were collected about demographic characteristics, women’s knowledge and attitude towards breastfeeding, antenatal and postnatal support and counseling, and feeding planning. Customized scales were utilized to assess knowledge and attitude. Factors associated with planning to breastfeed were reported.

**Results:**

660 women completed the survey questionnaire. The majority were 20 to 35 years of age, 10% were primiparous, and 30% were employed. 78% were knowledgeable about breastfeeding benefits and aware of WHO recommendations. 72% had a positive attitude towards breastfeeding. More than half received support from their husbands to breastfeed their infants, while less than 20% received any counseling from their obstetric providers. 97% reported their intention to breastfeed, and more than half indicated their willingness to breastfeed exclusively. With multivariable logistic regression modelling, predictors of EBF planning include: primiparity (AOR 1.79; 95% CI 1.1, 3.25), positive attitude (AOR 1.80; 95% CI 1.05, 3.1) and positive husband’s support (AOR 1.92; 95% CI 1.18, 3.15). Barriers include women’s employment (AOR 0.43; 95% CI 0.26, 0.70) and low birthweight (AOR 0.46; 95% CI 0.25, 0.84).

**Conclusion:**

Jordanian women are highly knowledgeable about breastfeeding benefits, and they exhibit a positive attitude towards breastfeeding resulting in a very high percentage intending to breastfeed their infants. Limited counseling about breastfeeding is a major gap in antenatal care. As intentions might not reflect the actual practice after delivery, gaps and barriers affecting the determinants of successful breastfeeding should be identified, and corrective tools should be implemented accordingly. Allocating a specific time for antenatal or postnatal counseling and support is expected to promote breastfeeding practice in our population.

## Background

The rate of exclusive breastfeeding (EBF) continues to decline in most countries despite the ongoing recommendation by the World Health Organization (WHO) and other international organizations to promote EBF practice for at least 6 months [1, 2]. Improving breastfeeding practice is considered an important global priority that has been included among the Millennium Development Goals established by the WHO in the past decade [1].

One of the main challenges in promoting EBF practice is related to maintaining breastfeeding for the recommended period, rather than its initiation after birth [3]. Among the reasons for low EBF rate is the limited knowledge women have about breastfeeding benefits, in addition to the limited support and encouragement they receive from their relatives or treating obstetricians [4, 5]. Also, the improvement in social and financial status has paradoxically contributed to the decline in the rate of EBF in certain cultures [6].

Women’s intention to breastfeed their infants is considered among the main predictors of EBF [7], and so exploration of the factors influencing their intention might help healthcare providers to address this issue during the antenatal care visits or after delivery in order to keep mothers motivated about breastfeeding.

Among the Ten Steps to successful breastfeeding included in the Baby Friendly Hospital Initiative (BFHI) is Step 3 which talks about the importance of informing all pregnant women about the benefits and management of breastfeeding [8, 9]. Accordingly, several studies have endorsed the importance of including a short session to review breastfeeding benefits during antenatal care visits to improve the breastfeeding rates among pregnant and newly delivered women. Mattar et al. have reported that a single session on breastfeeding education and counseling during the antenatal care visits might improve the breastfeeding practice for 3 months after birth [10]. Other reports highlighted that antenatal breastfeeding educational programs implemented directly with face to face counseling or by providing self-learning videos and written brochures, together with postnatal lactation support and counseling are regarded as important interventions to improve the rate of initiation and continuation of EBF, and to improve the overall breastfeeding practice [11].

Intention and planning of breastfeeding are related to the amount of knowledge and awareness women have antenatally, and these in turn, will be reflected on their attitude and subsequently their practice after delivery. Accurate evaluation of knowledge, awareness and attitude has been highlighted in breastfeeding-focused studies. Standardized scales have been implemented to assess feeding knowledge and attitude. The Iowa Infant Feeding Attitude Scale (IIFAS) was designed to measure women’s attitude towards breastfeeding and has been validated to be a reliable method for this purpose [12]. Similarly, the Infant Feeding Knowledge Test Forma A (AFORM) has been used as a valid and reliable tool to assess women’s knowledge and awareness about breastfeeding and its benefits [13].

In Jordan, exclusive breastfeeding was a traditional practice a few decades ago. More recently, trends of breastfeeding have changed among Jordanian women. Little research has been conducted in the past decade reviewing the rates and trends of breastfeeding. We reported a few years ago an EBF rate of 33% in infants aged 6 months compared with 87% rate of breastfeeding initiation after birth [14]. The practice of breastfeeding in our population manifested by high initiation rate after birth and significant decline in EBF by 6 months is comparable with the nearby Arab countries as reported from some local cross-sectional studies. For example, the rate of breastfeeding initiation and EBF at 6 months was reported to be 98 and 25% in United Arab Emirates compared with 57 and 19% in Qatar [15, 16]. Analysis of the factors that contribute to women’s attitude and planning should be targeted in breastfeeding studies and are expected to act as guidance for healthcare administration and policy making agencies in order to enhance EBF rates and to improve the overall breastfeeding practice. We, therefore conducted this questionnaire survey to assess the knowledge, awareness, motivation, attitude and planning of breastfeeding among Jordanian women in the antenatal and immediate postpartum periods, and to determine the factors associated with EBF.

## Methods

After obtaining approval from the Institutional review board at Jordan University of Science and Technology (IRB Number 505–2019), a face-to-face cross-sectional semi-structured questionnaire survey was distributed to two convenience samples of healthy women at three hospitals in the city of Irbid in North of Jordan during the period August 2019 to December 2019. One hospital, King Abdullah University Hospital (KAUH), is a tertiary academic center. The other two hospitals are a military hospital, and a public Ministry of Health-affiliated hospital. The three hospitals provide health service to more than two million of the Jordanian population with an annual number of deliveries exceeding 20,000. The majority of inhabitants in our district are middle class families with health insurance coverage through government and employment.

The first group of participants consisted of healthy pregnant women in the second or third trimester of gestation who presented for regular antenatal visit at the obstetric outpatient department. The second group included newly delivered women in the postpartum unit within 24 h after delivery. Participants were recruited from these two convenience samples until the target sample size was reached. All women who agreed to participate signed a written informed consent at the time of the interview. Women or infants who cannot breastfeed for medical reasons were excluded from the study. Among the excluded women were those with previous breast surgery and those receiving chemotherapy. Also excluded were infants with cleft palate or other major dysmorphism and infants with a family history of galactosemia among other siblings. The purpose of including two different groups of pregnant and newly delivered women was to get a better representative sample of our population. During data analysis, we did not intend to compare both groups. Unless there was a statistically significant difference between both subgroups in any of the variables or outcomes, both groups were combined into one group and data were accordingly analyzed and reported.

To examine the association between women’s planning to breastfeed with their awareness and knowledge, a Pearson correlation coefficient of 0.2 was used as an estimate for this relationship and assuming an 80% power and a 5% margin of error, a minimum sample size of 643 participants was needed to evaluate the women’s intention to EBF assuming a population-based rate of EBF around 50% with a 99% level of confidence.

The survey questions used for data collection were created using the IIFAS and the AFORM as the main references. These scales have been proven to be valid and reliable, and have been utilized in multiple breastfeeding-related studies [12, 13, 17]. We created our own customized scales to measure women’s knowledge and attitude by modifying some of the questions included in those reference scales to fit our population and culture. We have also combined some questions and changed the choices of the answers for other questions from a five-point Likert scale to answering a direct open-ended question.

Questions included data about maternal and newborn (for postpartum mothers) demographic characteristics. Knowledge and awareness were assessed using eight items with a yes/no answer for each item. Knowledge related items include awareness about WHO recommendations, definition of EBF, allowed vitamin supplements, role of breastmilk in infant health, the benefit of breastfeeding to protect mother against postpartum hemorrhage and certain malignancies, possible contraceptive effect of breastfeeding, improvement in breastmilk production, and breastfeeding with maternal sickness. Correct answers were awarded one point each. Any participant who achieved a total mark of four or more points (equivalent to 50% on the scale) was documented as knowledgeable.

Women’s attitude towards breastfeeding was assessed in a separate session utilizing a 10-item scale referencing the IIFAS, and they were offered a choice of agree vs disagree. These items include whether breastfeeding improves infant’s health and immunity, breastfeeding improves bonding, breastfeeding results in better infant’s weight gain, breastfeeding is considered the most ideal food, breastmilk is cheaper, breastmilk is easily digested with better tolerance, back to work is not a barrier against breastfeeding, breastfeeding convenience during social gathering, first feeding should be colostrum, and whether women prefer rooming in and agree with using infant formula. Each answer was awarded one mark if it favors breastfeeding and zero mark if it favors formula feeding. Any woman with a total score of five or more out of 10 was documented as having a positive attitude towards breastfeeding.

Another major part of the survey questionnaire focused on the support women receive from their obstetric care providers, husbands, other relatives, and work environment. Data were collected with direct open-ended questions selecting a yes or no answer. Planning and intention for feeding was assessed in another session utilizing the Infant Feeding Intentions scale as a guide, and women were accordingly categorized into three groups; exclusive breastfeeding (if they planned to breastfeed only without any supplement), mixed feeding or exclusive formula feeding. The reasons for mother preferences were also ascertained.

After a thorough training and final agreement on the study questionnaire by all authors, the created questions were translated from English to Arabic language, and then translated back into English version by a different person to ensure reliability and accuracy. After that, direct face-to-face interviews were conducted by two postgraduate physicians using both languages as needed. Answers were documented on the same questionnaire sheet for each participant. The study questionnaire was reassessed after a pilot review on a sample of 20 women, after which some more explanatory information was added to some of the questions related to knowledge and planning. Data were then transferred into an excel sheet and transcribed into different codes to facilitate data analysis.

### Statistical analysis

Data were analyzed using SPSS version 22. Continuous and categorical variables were presented using means and standard deviations (SD) or numbers and proportions as appropriate. Proportions of women who reported their intention to EBF were compared by each independent variable using chi-square distribution and *p* - values were reported accordingly. Variables with *p* - values of less than 0.25 were introduced into the logistic regression models. Backward logistic regression analysis was utilized to calculate the adjusted effect of independent variables on EBF intention. Adjusted odds ratios (AOR) and 95% Confidence Intervals (CI) were reported.

## Results

Six hundred and sixty women completed the survey questionnaire. Participants were equally distributed between both groups; 330 pregnant women in the antenatal care clinic and 330 newly delivered women in the postpartum unit. Table 1 summarizes the demographic characteristics of the women and their infants. The majority were between 20 and 35 years of age, 10% were primiparous, two thirds had experience with breastfeeding with their previous children in the past, and 30% were employed. Among women in the newly delivered group, 70% had C-section and nearly 30% of them had their infants admitted to the neonatal ICU.

**Table 1:** Maternal and infant characteristics

	**Total (*N*= 660)**	
	**Number**	***Percent***
Age (years) Mean ± SD (30.1 ± 5.8)		
less than 20	12	*1.8*
20 to 35	522	*79.1*
more than 35	126	*19.1*
Parity		
Primiparous	72	*10.9*
Multiparous	588	*89.1*
Previous BF experience		
None	207	*31.4*
Partial < 6 months	23	*3.5*
EBF < 6 months	119	*18.0*
Any BF > 6 months	311	*47.1*
Education		
< high school	103	*15.6*
high school	162	*24.5*
> high school	395	*59.8*
Employment Status		
No	467	*70.8*
Yes	193	*29.2*
Income (JOD)		
< 500	396	*60.0*
500 - 1000	230	*34.8*
> 1000	34	*5.2*
	**Total (330)**	
	**Number**	***Percentage***
Delivery		
Vaginal	102	*31*
C-section	228	*69*
Gestational age (weeks) Mean ± SD (38 ± 2.3)		
< 37	48	*15*
≥ 37	282	*85*
Gender		
Male	157	*48*
Female	173	*52*
NCIU admission		
No	235	*71*
Yes	95	*29*
Birthweight Mean ± SD (2942 ± 646)		
< 2500 grams)	69	*21*
≥ 2500 grams)	261	*79*

*BF* Breastfeeding, *EBF* Exclusive breastfeeding, *JOD* Jordanian dinars, *NICU* Neonatal intensive care unit

Women’s knowledge and attitude towards breastfeeding, as well as the support and counseling they received about its practice were summarized in Table 2. Around 78% were knowledgeable (received a score of four or more in the knowledge scale) about the benefits of breastfeeding and the WHO recommendations regarding breastfeeding practice. When asked about their knowledge and awareness regarding the benefits of breastfeeding, the most correctly selected answer was about the positive effect of breastfeeding on the infant immunity (85%). However, only 5% knew that EBF may have some contraceptive effect. Also, 72% had a positive attitude towards breastfeeding (received a score of five or more in the attitude scale) which was mostly reflected by their preference to have colostrum as the first feeding given to their infants and by reporting that breast milk is cheaper, and improves mother-infant bonding. Despite having a collectively positive attitude towards breastfeeding, 72% preferred not to have their infants stay with them in the postpartum ward all the time and agreed towards a mixed feeding pattern only during their stay in the newborn nursery or neonatal ICU.

**Table 2:** Knowledge, attitude and support

	Total (660)	
	Number	***Percent***
Knowledgeable (AFORM > 4*)		
No	144	*21.8*
Yes	516	*78.2*
Positive Attitude (IIFAS > 5©)		
No	182	*27.6*
Yes	478	*72.4*
Obstetric education		
No	538	*81.5*
Yes	122	*18.3*
Husband support		
No	298	*45.2*
Yes	362	*54.8*
Other Support		
None	78	*11.8*
Relatives	88	*13.3*
Healthcare	474	*71.8*
Work	11	*1.7*

*: Modified FORM A scale

©: Modified Iowa Infant Feeding Attitude Scale

Less than 20% of participants reported that their treating obstetrician talked to them about breastfeeding or encouraged them to breastfeed their infants. On the other hand, more than half indicated receiving support from their husbands.

As seen in Table 3, the majority of participants (97%) reported their intention to breastfeed and more than half indicated their plan to practice EBF after discharge from the hospital. More than 75% indicated their plan to continue breastfeeding for more than 6 months. The association between women’s plan to EBF and demographic factors, knowledge, attitude, and support is summarized in Table 4. After identifying factors associated with EBF, a backward logistic regression model was applied to determine predictors and barriers. The predictors of EBF include primiparity (AOR 1.79; 95% CI 1.1, 3.25), positive attitude (AOR 1.80; 95% CI 1.05, 3.1) and positive husband’s support (AOR 1.92; 95% CI 1.18, 3.15). Barriers include women’s employment (AOR 0.43; 95% CI 0.26, 0.70) and low birthweight (AOR 0.46; 95% CI 0.25, 0.84). See Table 5.

**Table 3:** Intention and planning

	Total (660)	
	Number	Percent
Feeding plan		
BF only	352	53.3
Mixed	289	43.8
Formula	19	2.9
Breastfeeding duration		
Never	19	2.9
1 month	8	1.2
1–6 months	123	18.6
> 6 months	510	77.3

*BF* Breastfeeding

**Table 4:** Planning to EBF according to social characteristics, knowledge, attitude and support

	EBF (***n*** %)				***P - value***
	No		Yes		
Age (years)					
< 20	4	*33.3%*	8	*66.7%*	*0.135*
20 to 35	236	*45.2%*	286	*54.8%*	
≥ 35	68	*54.0%*	58	*46.0%*	
Parity					
Primiparous	23	*31.9%*	49	*68.1%*	*0.005*
Multiparous	285	*48.5%*	303	*51.5%*	
Previous BF experience					
None	89	*43.0%*	118	*57.0%*	*0.266*
Partial < 6 months	13	*56.5%*	10	*43.5%*	
EBF < 6 months	63	*52.9%*	56	*47.1%*	
Any BF > 6 months	143	*46.0%*	168	*54.0%*	
Education					
< high school	38	*36.9%*	65	*63.1%*	*0.004*
high school	65	*40.1%*	97	*59.9%*	
> high school	205	*51.9%*	190	*48.1%*	
Employment status					
No	181	*38.8%*	286	*61.2%*	*0.000*
Yes	127	*65.8%*	66	*34.2%*	
Income (JOD)					
< 500	162	*40.9%*	234	*59.1%*	*0.000*
500–1000	121	*52.6%*	109	*47.4%*	
> 1000	25	*73.5%*	9	*26.5%*	
Delivery mode					
VD	49	*48.0%*	53	*52.0%*	*0.360*
C-S	116	*50.9%*	112	*49.1%*	
Gestational age (weeks)					
< 37	27	*56.3%*	21	*43.8%*	*0.218*
≥ 37	138	*48.9%*	144	*51.1%*	
Gender					
Male	77	*49.0%*	80	*51.0%*	*0.413*
Female	88	*50.9%*	85	*49.1%*	
Birthweight (grams)					
< 2500	41	*59.4%*	28	*40.6%*	*0.05*
≥ 2500	124	*47.5%*	137	*52.5%*	
NICU admission					
No	119	*50.0%*	119	*50.0%*	*0.514*
Yes	48	*50.5%*	47	*49.5%*	
Knowledgeable					
No	72	*50.0%*	72	*50.0%*	*0.208*
Yes	236	*45.7%*	280	*54.3%*	
Positive attitude					
No	103	*56.6%*	79	*43.4%*	*0.001*
Yes	205	*42.9%*	273	*57.1%*	
Obstetric education					
No	250	*46.5%*	288	*53.5%*	*0.454*
Yes	58	*47.5%*	64	*52.5%*	
Husband support					
No	153	*51.3%*	145	*48.7%*	*0.018*
Yes	155	*42.8%*	207	*57.2%*	
Other Support					
None	43	*55.1%*	35	*44.9%*	*0.106*
Relatives	34	*38.6%*	54	*61.4%*	
Healthcare	222	*46.8%*	252	*53.2%*	
work	3	*27.3%*	8	*72.7%*	

*BF* Breastfeeding, *EBF* Exclusive breastfeeding, *JOD* Jordanian dinars, *NICU* Neonatal intensive care unit, *VD* Vaginal delivery, *C-S* cesarean section

**Table 5:** Multivariable logistic regression analysis of factors associated with EBF

	*P-value*	AOR	95 % CI	
			Lower	Upper
Parity				
Primiparous	*0.05*	1.79	1.1	3.25
Multiparous		1		
Employment status				
No		1		
Yes	*0.001*	0.43	0.26	0.70
Birthweight (grams)				
< 2500	*0.012*	0.46	0.25	0.84
≥ 2500		1		
Positive attitude				
No		1		
Yes	*0.033*	1.80	1.05	3.10
Husband support				
No		1		
Yes	*0.009*	1.92	1.18	3.15

*EBF* exclusive breastfeeding, *AOR* Adjusted odds ratio, *CI* Confidence interval

## Discussion

In this local multi hospital-based sample of pregnant and newly delivered women, we reported that Jordanian women are highly knowledgeable about the benefits of breastfeeding, aware of the WHO recommendations, exhibit a positive attitude towards breastfeeding, and the majority plan on breastfeeding their infants for at least 6 months.

Decisions around breastfeeding practice are affected by multiple factors including knowledge, awareness, attitudes and motivation. In our cohort, when participants were asked about the benefits of breastfeeding, most of them reported spontaneously that breast milk significantly contributes to better infant immunity and less risk of infections even before directly asking them to answer that question. Several other studies that focused on assessing women’s knowledge about the benefits of breastfeeding concluded that women have excellent knowledge regarding certain aspects of breast milk benefits, mainly its impact on immunity and good infant health outcomes [18]. Moreover, Raissian et al. reported that prenatal intention was considered a strong factor associated with a better infant’s health even if infants were not breastfed [19]. When compared to studies that explored breastfeeding knowledge and attitude in other Arab countries, our findings were very promising. The major gap in women’s knowledge about breastfeeding in certain Arab cultures is related to their perception that breast milk might not contain adequate nutrition, and this is the major reason to start supplementing with infant formula. This perceived belief has been highlighted in previous studies from the Middle East region [20, 21]. This misconception was found to be less obvious in older mothers with previous experience with breastfeeding [22]. In their analysis from Lebanon, Osman et al. reported that certain cultural beliefs, such as “breastmilk contains inadequate nutrients and might result in infantile colic or even causing harm to infants”, have a major negative impact on breastfeeding and were associated with early introduction of formula. The authors concluded that culture-specific counseling should be implemented to improve breastfeeding practice [20]. Some knowledge related studies about breastfeeding in the Arab world targeted college students in healthcare sections and reported variable results according to the item tested [23, 24]. For example, in Egypt, analysis of breastfeeding knowledge and attitude among nursing students revealed unexpectedly low scores highlighting the importance of implementing programs that focus on breastfeeding during the undergraduate study programs [23]. A similar finding was identified among Syrian and Lebanese students enrolled in health-related undergraduate studies by Hamade et al. [24].

With a positive attitude, women acquire a great motivation, and this will be reflected in their intentions and practices. Our finding of a positive association between positive attitude and planning to breastfeeding is consistent with several studies from USA and UK [25, 26]. Therefore, it is believed that promoting the best breastfeeding practices should start a long time before delivery and should be included in the antenatal education and care. However, the attitude towards breastfeeding might be negatively influenced by local hospital set-up in resource limited countries like Jordan and other nations in our region. The BFHI was established by the WHO and UNICEF in the early 1990’s to improve the rate of exclusive breastfeeding [8]. However, there has been no consistent reporting about its impact on the rate of EBF among different institutions and countries. With the busy clinic schedule and labor wards, the lack of healthcare education together with the absence of baby friendly units in our hospitals could have contributed to this trend. Such limited resources act against the BFHI particularly Step 5 which encourages providing enough support for lactating mothers to overcome all obstacles against successful initiation and maintain breastfeeding [8]. In addition, although our participants indicated their preference to have their babies receive colostrum as their initial feeding, a good percentage preferred to have their infants stay in the newborn nursery and agreed with using infant formula at times. This finding is a clear example of an educational gap in applying Steps 4 and 7 of the BFHI which encourage skin-to-skin care and initiating breastfeeding immediately after birth and reinforce the practice of rooming-in together [8]. Similarly, Ogbonna 2009 and Shaker 2004 have reported that lack of consistent education by the hospital staff was a strong barrier to exclusive breastfeeding [22, 27]. Other reports concluded that the education process is an ongoing task and should continue after home discharge to avoid early cessation of breastfeeding [28].

Besides its association with a positive women’s attitude, we reported that the intention to breastfeed was significantly determined by the support women receive towards breastfeeding practice. Research has repeatedly found that women’s pre-birth breastfeeding intention is a good predictor of the actual duration of breastfeeding [7]. Regardless of women’s beliefs and planning, breastfeeding practice is highly dependent on the amount of support they receive from their husbands, healthcare providers, relatives, friends, work environment and hospital staff [11]. In our cohort, husband’s support was a major predictor for breastfeeding, while limited support and counseling by the obstetric care provider was reported by most of our participants as a major concern. Our findings are consistent with several other studies including systematic reviews and meta-analysis reports [29, 30]. In their systematic review of professional support interventions for breastfeeding, Hannula et al. reinforced the importance of providing obstetric professionals with educational tools to support breastfeeding which would in turn improve the women’s self-efficacy and power to ensure better breastfeeding behavior [30]. In their recently published study, Van Dellen et al. examined the association between breastfeeding practice and a comprehensive evidence based intervention named “Breastfeeding Support Program (BSP)” that combines antenatal and postpartum support and education, and concluded that BSP promotes longer duration and higher exclusivity of breastfeeding among their participants [31]. Besides obstetric care providers, neonatal care providers including neonatologists and nurse practitioners should frequently receive enough education and reminders to support breastfeeding to ensure compliance with Step 2 of the BFHI [8]. Studies have shown that discussing the benefits of breastmilk during prenatal consultations performed by the neonatologist for pregnant women with potential premature delivery would result in a higher rate of providing breastmilk for infants admitted to the NICU during and after hospital stay [32]. Additionally, the strong association between husband’s support and intention to breastfeed among the women in our culture reflects a strong adherence to Step 3 of BFHI and makes us believe that husbands should attend the antenatal and postnatal educational sessions about breastfeeding as their influence might have a great impact on their wives’ intentions [8].

One of the main challenges in promoting EBF practice is related to maintaining breastfeeding for the recommended period rather than its initiation after birth [28]. The effect of women’s education and employment status on breastfeeding practice has been inconsistent [33]. The rate of employed women in our studied population was 30%. The majority of them answered “Going back to work after short maternity leave with limited support and space to breastfeed our infants while at work” as the major reason against exclusive breastfeeding. Our finding regarding women’s concern about breastfeeding in the work environment is in agreement with several other reports from the region [14, 16, 21]. The short maternity leave and the lack of supportive measures in the workplace, such as the lack of day care centers and the inability of having multiple breaks during the daytime to breastfeed their infants, discourage mothers from EBF and push them to introduce infant formula. On the other hand, Wallenborn 2019 analyzed US data from Infant Feeding Practices Survey II and reported that work environment support was strongly associated not only with a higher breastfeeding rate, but also with a positive self-efficacy and a longer duration of breastfeeding [34]. Although breast pumps are not widely available, there has been an increasing interest of using them among Jordanian women and this might be a solution to help employed mother feed their infants expressed milk while they are away at work.

Despite the challenges that might interfere with breastfeeding, our report finding of high women’s knowledge and positive attitude are promising determinants for improving the breastfeeding practice in Jordan.

This study is not without limitations. The participants in our study who were selected from two different convenience groups represent the inhabitants of our region who mostly belong to the middle socio-economic group only. A population-based study including random selection from higher and lower socio-economic groups will give more accurate data to represent the whole Jordanian population. Also, being a cross-sectional study, it is important to mention that the reported rates in this study reflect women’s planning towards breastfeeding and might not reflect the actual rate of breastfeeding given the potential limitations that women might encounter against breastfeeding with time. Another limitation is the modification of our questionnaire from the standard scales about knowledge and attitude that have been tested for validity and reliability, which precludes direct comparison with other studies. However, this might be a point of strength about our study since we modified those reference scales and created customized scales that seem more applicable and easily presented among our study participants.

## Conclusion

In conclusion, it is crucial to determine the factors that contribute to women planning to breastfeed their infants, so that targeted counseling programs and motivation strategies are established to improve the breastfeeding practice. There is strong evidence that high knowledge scores and positive attitudes are among the major determinants of successful breastfeeding practice in different cultures and so culture-specific policies should be established to identify gaps and barriers affecting these determinants in order to implement the corrective tools. Appropriate public health policies to help mothers breastfeed for at least 6 months, and to remove the barriers to breastfeeding, will be required to meet the WHO recommendations. The rate of breastfeeding initiation after birth is high in Jordan while EBF by 6 months is far below the WHO recommendation. This necessitates focusing on identifying the obstacles that women face during the breastfeeding journey and promoting culture-specific solutions that address these challenges better and offer women the options for better adaptation.

Focusing on antenatal educational programs as well as healthcare provider support and counseling should be included as essential components in the antenatal and postnatal care to improve the practice of breastfeeding in Jordan and among other cultures in the region. Also, applying better employment policies that give women more flexibility to breastfeed their infants is considered to be important. We recommend that policymakers implement policies that adhere to the BFHI and Ten Steps to successful breastfeeding. With advances in technology, utilizing social media resources could be helpful in achieving these goals.

## Data Availability

The datasets used and analyzed during our study are available from the corresponding author upon reasonable request.

## Abbreviations

EBF: Exclusive Breastfeeding
WHO: World Health Organization
AAP: American Academy of Pediatrics
IIFAS: The Iowa infant feeding attitude scale
AFORM: The Infant Feeding Knowledge Test Forma A
IRB: Institutional Review Board
BFHI: Baby Friendly Hospital Initiative
